# Surface–Atmosphere Moisture Interactions in the Frozen Ground Regions of Eurasia

**DOI:** 10.1038/srep19163

**Published:** 2016-01-18

**Authors:** Trent W. Ford, Oliver W. Frauenfeld

**Affiliations:** 1 Department of Geography and Environmental Resources, Southern Illinois University, Carbondale, IL 62901; 2 Department of Geography, Texas A&M University, College Station, TX 77843-3147.

## Abstract

Climate models simulate an intensifying Arctic hydrologic cycle in response to climatic warming, however the role of surface-atmosphere interactions from degrading frozen ground is unclear in these projections. Using Modern-Era Retrospective Analysis for Research and Applications (MERRA) data in high-latitude Eurasia, we examine long-term variability in surface-atmosphere coupling as represented by the statistical relationship between surface evaporative fraction (EF) and afternoon precipitation. Changes in EF, precipitation, and their statistical association are then related to underlying permafrost type and snow cover. Results indicate significant positive trends in July EF in the Central Siberian Plateau, corresponding to significant increases in afternoon precipitation. The positive trends are only significant over continuous permafrost, with non-significant or negative EF and precipitation trends over isolated, sporadic, and discontinuous permafrost areas. Concurrently, increasing EF and subsequent precipitation are found to coincide with significant trends in May and June snowmelt, which potentially provides the moisture source for the observed enhanced latent heating and moisture recycling in the region. As climate change causes continuous permafrost to transition to discontinuous, discontinuous to sporadic, sporadic to isolated, and isolated permafrost disappears, this will also alter patterns of atmospheric convection, moisture recycling, and hence the hydrologic cycle in high-latitude land areas.

Earth’s surface partitions incoming energy into the latent and sensible heat flux, which modifies near surface temperature^1^,^2^,^3^, the development of clouds^4^,^5^, and the potential for convective precipitation^6^. Soil moisture can impact atmospheric conditions primarily through its modulation of surface energy and moisture flux^7^, making the partitioning of latent and sensible heat flux crucial for land-atmosphere interactions^5^,^8^. Enhanced soil moisture results in more latent than sensible heating, which generally depresses air temperature and increases atmospheric humidity. In contrast, drier than normal soils force enhanced sensible heating, and increased air temperature.

Soil moisture, surface energy flux, and precipitation data are most readily available in Europe and North America, therefore the majority of studies examining land-atmosphere interactions have investigated these regions. However, climate change in high latitude areas^9^,^10^, coupled with large-scale thawing of permafrost and seasonally frozen ground in the Eurasian high-latitudes^11^,^12^,^13^,^14^ potentially also make regions such as central–eastern Russia subject to amplified land-atmosphere interactions. Decreased albedo in arctic Alaska, due to thawing permafrost and vegetation expansion, contributes to increasing temperatures in this region^15^. Concurrently, increasing evapotranspiration (ET) over the Lena watershed in Siberia is attributable to more moisture availability in the surface and subsurface^16^. Based on the limited literature, land-atmosphere feedbacks are likely relevant to high latitude hydroclimatology; however, due to sparse observations, land-atmosphere interactions in this region have not been extensively investigated. In this study, we therefore examine long-term variability and trends in surface-atmosphere relationships. We hypothesize the presence or absence of permafrost contributes to altered surface energy and moisture fluxes, ultimately capable of influencing precipitation and the hydrologic cycle. Permafrost represents an impermeable barrier to moisture, resulting in a saturated or near-saturated surface layer in many continuous and discontinuous permafrost zones. Moisture from snowmelt, precipitation, and from the soil itself therefore cannot drain away. These surface conditions should lead to enhanced local recycling of moisture^17^, convective cloud cover, and, potentially, precipitation during the warm season. In warmer subarctic regions, permafrost is discontinuous or absent, therefore soils can be quite dry because infiltration is not restricted. The expectation, therefore, is that in the warm season in areas of continuous permafrost, a saturated active layer should result in greater atmospheric moisture and higher likelihood of precipitation, compared to sporadic and isolated permafrost and seasonally frozen ground regions.

To test the role of frozen ground on atmospheric moisture, we analyze surface energy fluxes as represented by surface evaporative fraction (EF). EF is the ratio of latent heat (LH) to latent plus sensible heat (SH) (equation 1) or, simply, the ratio of latent heating to the available energy at the surface:


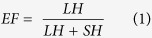


This study focuses on monthly and annual variability in EF as well as long-term EF trends in Eurasia. We use logistic regression to quantify the statistical coupling between EF and precipitation over this region. We then relate EF variability and trends to permafrost distribution.

## Data & Methods

### MERRA

Because of the limited *in situ* data availability, we use LH, SH, and precipitation from the Modern-Era Retrospective analysis for Research and Applications (MERRA) Land dataset, a reanalysis with a data assimilation scheme that is focused specifically on correctly simulating the hydrologic cycle. MERRA uses the Goddard Earth Observing System version 5 (GEOS-5^18^) for data assimilation. A recent evaluation of atmospheric reanalysis products revealed MERRA, in general, has the highest correlation with observations^19^. However, it should be noted that because of model biases and observational inconsistencies, all reanalyses have potential drawbacks^20^,^21^. In addition, MERRA has been shown to be susceptible to changes in observation systems, attributable to the introduction and removal of various satellite remote sensing-based data^18^. In response to these issues, MERRA precipitation forcings were adjusted toward observations from the Global Precipitation Climatology Project^22^. This resulted in the more realistic MERRA-Land surface hydrology products, which we therefore use in this study. Additional caveats for reanalysis-based approaches such as ours are the potential for land-atmosphere coupling that is partly attributable to model physics and not solely to actual land surface feedbacks. Finally, reanalyses have potential limitations in simulating land surface feedbacks within moisture-limited and energy-limited or atmospherically controlled conditions^23^. The results and conclusions from this study should therefore be interpreted with these caveats in mind. Nonetheless, reanalysis products such as MERRA represent the only source of data that is continuous in both time and space in data-sparse regions such as the Arctic.

We use hourly data from the MERRA-Land product^24^, which has a 1/2° × 2/3° spatial resolution, and focus on 1979–2012 over the Eurasian high latitudes, defined here as the land areas north of 50°N and from 15°E–165°W. Hourly latent and sensible heat flux between 0600 and 1200 LST were extracted, from which morning EF is calculated at the hourly time-step and then averaged to daily resolution. EF is strongly influenced by incoming energy and moisture availability^25^, and therefore varies spatially. To account for this spatial variability, we convert EF values to anomalies by subtracting the monthly means. Daily EF anomalies are related to afternoon precipitation in the form of cumulative totals, summed daily between 1300 and 2000 LST. MERRA-Land precipitation data are strongly correlated with observations in the Arctic^19^, and are considered appropriate for use in our study. In addition to morning EF and afternoon precipitation, we examine daily snow cover (% grid cell) from the MERRA-Land dataset. Monthly and annual trends in EF, precipitation, and snow cover are determined using the Mann-Kendall trend test, and are assessed for statistical significance at the 95% confidence level.

### Permafrost Distribution

Permafrost is a major subsurface feature across the high latitudes, and has been shown to impact moisture availability for groundwater and discharge^26^. Therefore the presence or absence of permafrost is an important influence on high-latitude hydrological processes^27^. To evaluate whether permafrost distribution could influence EF and ultimately feedback to modify the atmosphere, we use the Permafrost Zonation Index (PZI)^28^. The PZI is derived from a global model of permafrost extent using high-resolution elevation and temperature datasets. PZI values range from 0–100, and we categorize them to correspond with the International Permafrost Association’s permafrost classes from the comprehensive Circum-Arctic Map of Permafrost and Ground-Ice Conditions^29^, such that 90–100% is continuous permafrost, 50–90% is discontinuous, 10–50% is sporadic, and <10% represents isolated permafrost (Supplementary Fig. S1).

### Logistic Regression

Land–atmosphere feedbacks as they pertain to precipitation are difficult to quantify, due in part to the temporal autocorrelation of precipitation^30^ and the various time lags at which variables such as soil moisture and EF modify atmospheric conditions leading to or diminishing precipitation. Simple correlation or ordinary least squares regression do not account for either of these issues, making interpretation problematic. Therefore, we focus on the connection between morning EF and the probability of afternoon precipitation by treating precipitation events as a binary variable (1 = precipitation, 0 = no precipitation) and use logistic regression^31^ to describe the probabilistic relationship between EF and precipitation. Logistic regression uses the independent variable, EF, to predict the logit transformation of the dependent variable, precipitation. The output is the odds ratio of the dependent variable. Here, the logistic regression model estimates the probability of afternoon precipitation given a morning EF anomaly. Specifically, hourly 1300–2000 LST precipitation is summed, and then converted into a binary variable, with 1 representing precipitation over 3 mm, and 0 representing accumulation less than 3 mm. An absolute precipitation accumulation threshold was used instead of precipitation anomalies, to eliminate trace precipitation events that could artificially inflate the regression model goodness-of-fit statistics. Similar studies using probabilistic models have also used absolute precipitation as their event indicator^6^,^32^. The daily morning EF anomalies and corresponding precipitation occurrences are then used to build the logistic regression model, from which the probabilistic relationship between the two variables can be determined. We do not attempt to interpret the output of the logistic regression model in terms of the linear relationship between EF and precipitation accumulation. This is because the intensity of convective precipitation and total precipitation accumulation are largely determined by non-land surface controls such as large-scale moisture convergence and free tropospheric moisture content^6^. Instead, the logistic regression model will give us a probabilistic relationship between surface flux and precipitation. Similar to^33^, the logistic regression model takes the form of:


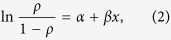


where 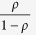
is the odds ratio of the occurrence of precipitation, *α* is the Y-intercept and *β* is the regression coefficient. The *β* value from the model is transformed into the probability of afternoon precipitation greater than 3 mm, given a 1-unit increase in EF anomalies. In this case, one unit equates to an increase of 0.01 in morning EF. The model output is the log odds ratio of afternoon precipitation occurrence and the slope of the regression represents the relationship between the two variables. For example, when the slope is positive, an increase in morning EF would increase the odds of precipitation occurring in the afternoon. The significance of the logistic regression was assessed using Wald’s chi-squared test^33^.

## Results

### Surface EF

**Figure 1:**
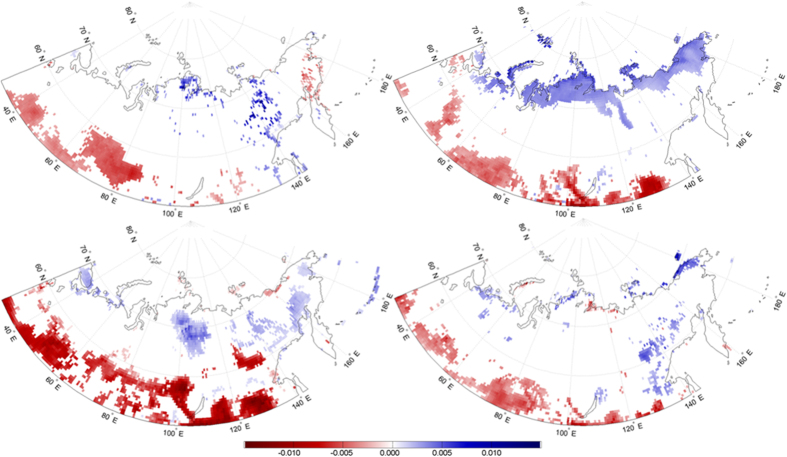
Surface EF trends (yr^−1^) from monthly MERRA EF in (**a**) January, (**b**) April, (**c**) July, and (**d**) October. Trends are evaluated over the period 1979 to 2012. Maps were generated using Matlab software.

**Figure 2:**
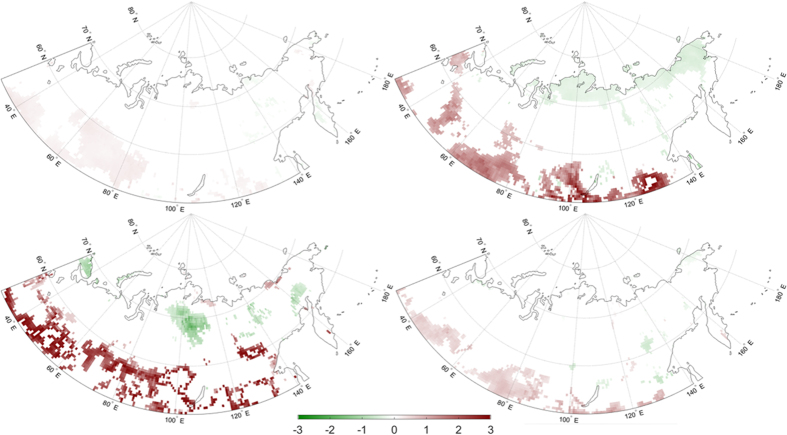
Sensible heat flux trends (W m-2 yr-1) from monthly MERRA SH in (**a**) January, (**b**) April, (**c**) July, and (**d**) October. Trends are evaluated over the period 1979 to 2012. Maps were generated using Matlab software^37^.

**Figure 3:**
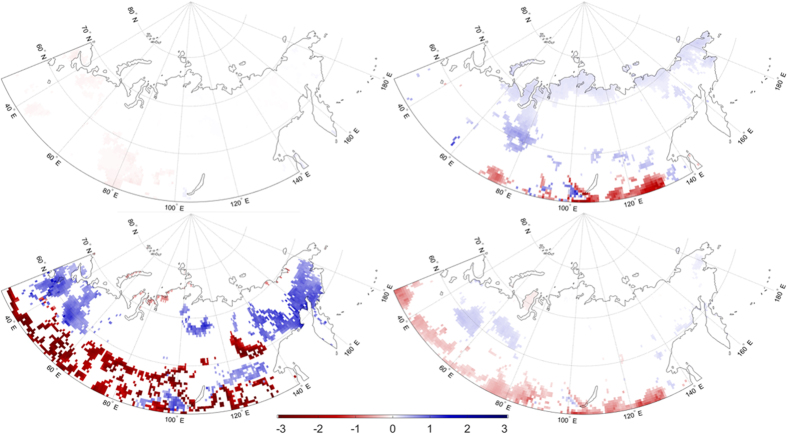
Latent heat flux trends (W m-2 yr-1) from monthly MERRA LH in (**a**) January, (**b**) April, (**c**) July, and (**d**) October. Trends are evaluated over the period 1979 to 2012. Maps were generated using Matlab software^37^.

Trends in 1979–2012 monthly EF are calculated using the Mann-Kendall trend test. The patterns of statistically significant surface EF trends are shown for the mid-season months of January, April, July, and October in Fig. 1. Morning EF trends in the southern half of the study region are consistently negative, with strongest trends in January. Less extensive patches of positive EF trends occur in the north-central region of Eurasia in April and July, but are not evident in October or January. To help interpret these trends, we separate surface EF into its LH and SH components, and assess the significant trends over the same time period (Figs 2 and 3). The negative surface EF trends in January do reflect long-term changes in LH and SH in this region of Siberia; however, the absolute values of these changes are several orders of magnitude smaller than for the other months, attributable to very small LH and SH fluxes in January (i.e., limited winter energy). In April, however, the southern half of the study region shows strong, positive SH trends, but no significant changes in LH. This suggests the negative April EF trends in this part of Siberia are primarily attributable to increased SH. Positive April surface EF trends in the northern part of Eurasia are, in contrast, a function of both decreased SH and increased LH. July negative surface EF trends in the southern half of the study region correspond with both increased SH and decreased LH. Concurrently, an area of strong, positive July surface EF trends over the Central Siberian Plateau, approximately 90°–115°E and 65°–72°N, is attributable more to decreased SH than to increased LH, although both are apparent in this region. Finally, relatively small decreases in October surface EF coincide with increased SH and decreased LH.

Based on the EF, LH, and SE trends (Figs 1, 2, 3), concurrent decreases in LH and increases in SH in the southern half of the study region have led to decreased surface EF. Meanwhile, smaller regions of positive surface EF trends in April and July are consistent with decreased SH and, to a lesser extent, increased LH in those areas.

### Precipitation

**Figure 4:**
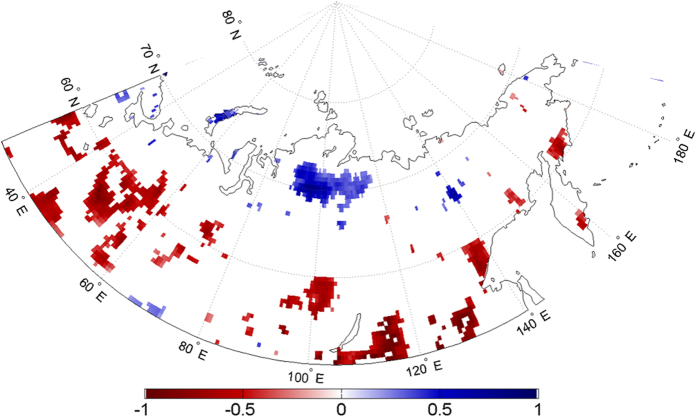
July precipitation trends (mm yr^−1^) from MERRA afternoon precipitation. Trends are calculated over the period 1979–2012. Maps were generated using Matlab software^37^.

Similar to surface EF, we calculate trends in afternoon precipitation accumulation for each month over the study region between 1979 and 2012. The only month showing any significant precipitation changes is July, when sporadic negative precipitation trends are evident across the southern part of the region. Also evident is an extensive region of significant precipitation increases over the Central Siberian Plateau (Fig. 4). The region of increased July afternoon precipitation coincides with significant increases in July surface EF, suggesting a potential association.

**Figure 5:**
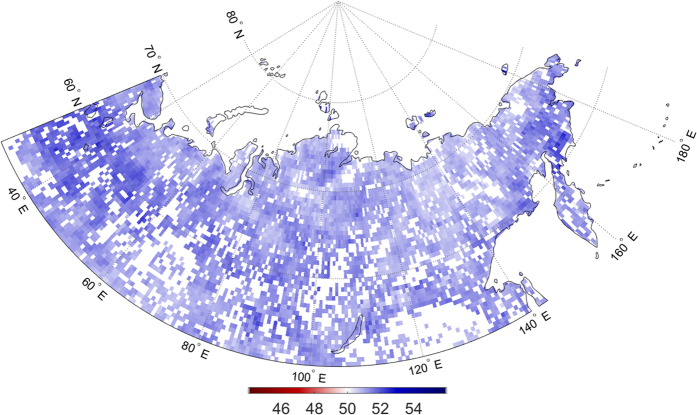
Probability of afternoon precipitation (%) in July corresponding with a 1-unit (0.01) increase in morning surface EF. Colored grid cells show a significant relationship between morning EF and the probability of afternoon precipitation, as evaluated by a logistic regression model. Maps were generated using Matlab software^37^.

The probability of afternoon precipitation, given this increase in morning EF anomalies is shown in Fig. 5. All grid cells with a statistically significant model fit show a probability greater than 50%, meaning that for all colored grid cells, an increase in EF anomalies the morning results in an increase in the probability of afternoon precipitation. The probability values over the continent range from 0.505 to 0.540, representing 0.5% to 4.0% increases in probability. Although these changes may seem low, they can be considerable given the annual trends in EF anomalies (Fig. 1).

**Figure 6:**
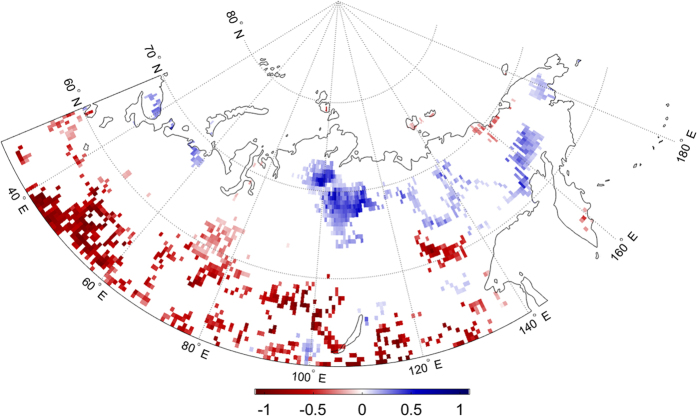
Annual change in probability (%) of July afternoon precipitation as a function of surface EF. Maps were generated using Matlab software^37^.

To test this, we substitute the EF increase with the observed annual EF trends from Fig. 1, to obtain the actual change in afternoon precipitation probability accounted for by increased/decreased EF anomalies. The patterns of these probability changes are shown in Fig. 6, where positive (blue) changes represent increased precipitation probabilities and negative (red) changes are decreased probabilities. The statistical relationship between morning EF and afternoon precipitation (Fig. 5) determined by the logistic regression is relatively consistent across the continent. Therefore, the locations and signs/magnitudes of probability changes are primarily controlled by the sign/strength of EF trends. Consequently, the Central Siberian Plateau region shows a 0.5% to 1% annual increase in the probability of afternoon precipitation, attributable to positive EF trends (0.009 yr^−1^). It is worth noting these trends are EF anomaly trends, explaining the relatively small, yet significant magnitude. Concurrently, the areas to the south showing negative EF trends exhibit decreased precipitation probability on the order of −0.5% to −1%.

### Permafrost and Snow Cover

**Figure 7:**
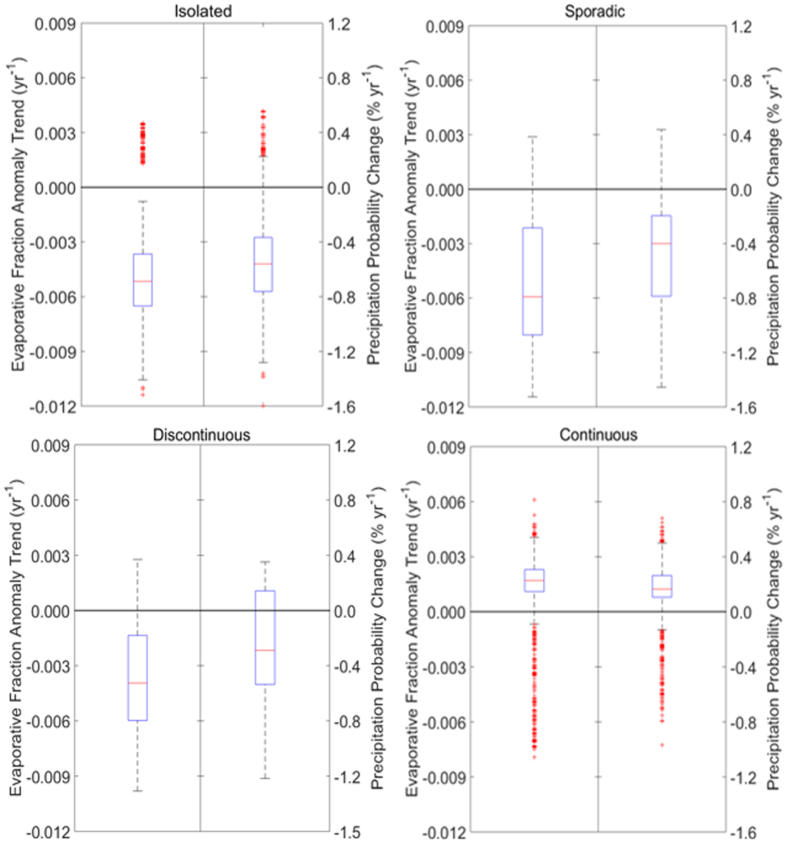
Evaporative fraction anomaly trends and corresponding change in precipitation probability, composted by underlying permafrost class. The evaporative fraction anomaly trends are shown in the left boxplot in each panel, and the change in precipitation probability is shown in the right boxplot. Permafrost information is taken from the Permafrost Zonation Index PZI).

The statistical relationship between morning EF and the probability of afternoon precipitation in July, as demonstrated with the logistic regression, suggest observed trends in EF are potentially related to precipitation over the same region. However, attributing changes in surface EF is difficult, as several moisture sources can potentially influence monthly and annual EF variability. While atmospheric advection represents a majority of the moisture source, precipitation recycling accounts for up to 28%, with maxima occurring during July in Eurasia^17^. Furthermore, both absolute EF values (Supplementary Fig. S2) and EF trends (Fig. 1) show some latitudinal variability, reflecting either a potential coupling with latitude-varying parameters such as permafrost and snow cover, or simply reflecting the proximity to the ocean. We examine the potential for permafrost and snow cover to contribute to the observed changes in July surface EF across the Eurasian domain displayed in Fig. 1. We composite both July EF anomaly trends and the changes in the probability of July afternoon precipitation by permafrost type using the PZI (Fig. 7). The mean July EF trend and July precipitation probability change in the continuous permafrost zone are positive, and both are statistically significantly different from the other permafrost categories based on a Student’s t-test at the 95% confidence level. The distributions of EF trends and precipitation probability for discontinuous permafrost exhibit large ranges, with both positive and negative values that suggest a transitional nature of this permafrost zone, where some areas (likely underlain by permafrost) exhibit positive values, while the permafrost-free zones are likely characterized by negative EF trends and precipitation probability changes. In both sporadic and isolated permafrost regions, EF trends and precipitation probability changes are negative and not statistically significantly different between these two categories. The permafrost composites of EF trends and precipitation probability changes suggest differential energy partitioning and different atmospheric responses corresponding to permafrost classes. Furthermore, it indicates both increased atmospheric moisture and increased precipitation in continuous permafrost, with opposite trends for isolated and sporadic zones. However, it is important to note that both EF trends and permafrost classes similarly vary with latitude, which could also account for these relationships.

**Figure 8:**
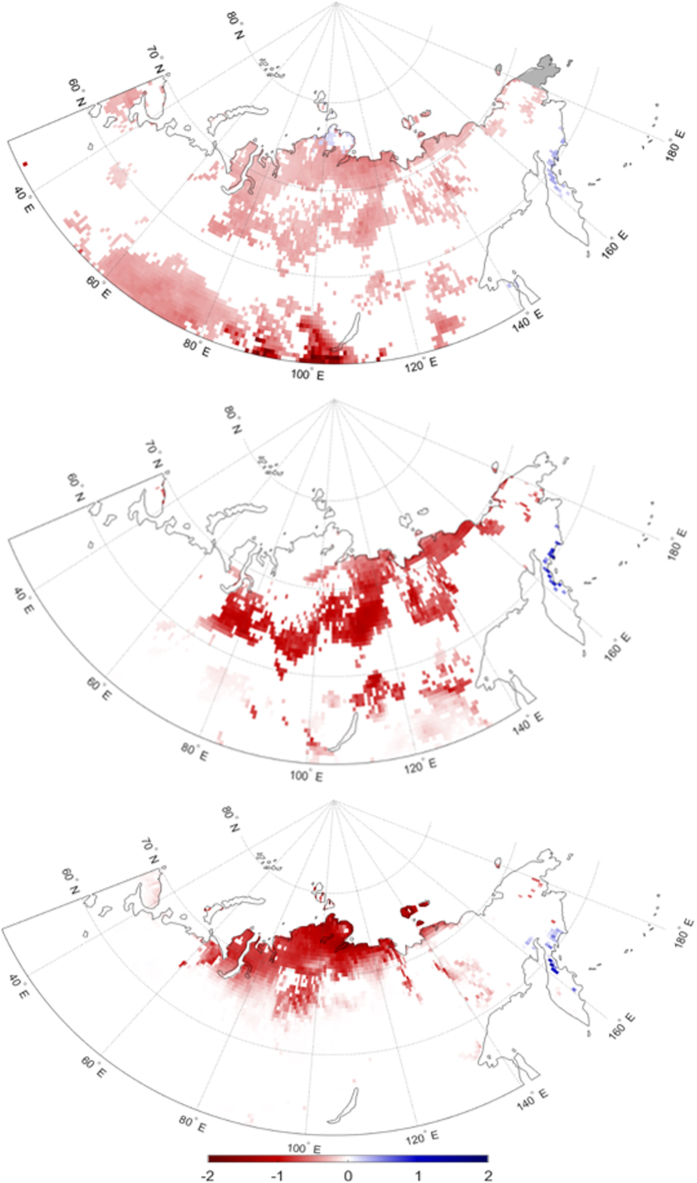
Significant 1979–2012 trends in (**a**) the first day of the year with less than 50% snow cover by grid cell, (**b**) percent of snow cover in May, and (**c**) July. Maps were generated using Matlab software^37^.

Changes in snow cover could also be related to EF trends, and we therefore examine changes in snow cover (% grid cell) between 1979 and 2012. We first quantify the overall change in snow cover by noting the first day of each year when less than 50% of the grid cell is snow covered. Extensive areas in both the north-central and south-central portions of the continent exhibit statistically significant negative trends in the first day with less than 50% snow cover (Fig. 8a). In general, snow is melting earlier in the year over these regions at a rate of 0.5–2 days per year. July snow cover is confined to the farthest north-central part of the study region (Supplementary Fig. S3). In addition, snow cover over the Central Siberian Plateau is rare in July, and typically does not exceed 1 mm on July 1^st^. Therefore, we focus on changes in snow cover leading up to July. Snow cover trend maps in May and June (Fig. 8) show significant negative trends (earlier snow melt) in the Central Siberian Plateau region, where positive EF and precipitation trends are observed in July. It is thus plausible that earlier snow melt in May and June in the Central Siberian Plateau could result in enhanced moisture availability at the surface in July. Wetter soils and more moisture at the surface, in combination with permafrost inhibiting drainage, increases EF through enhanced LH, which could feedback to precipitation, as observed in this study. However, further investigation is necessary to demonstrate a definitive physical linkage between earlier snowmelt and increased surface EF in this part of Eurasia.

## Summary and Conclusions

We hypothesize the presence/absence of permafrost may contribute to altered surface energy and moisture fluxes, ultimately capable of influencing precipitation. To test this, we analyzed MERRA-Land data over 1979–2012 to examine patterns and trends in surface EF and precipitation over the Eurasian high latitudes. We found significant negative surface EF trends in the southern half of the study region, attributable to increased SH with little to no appreciable change in LH. In contrast, the northern-central portion of the study region showed significant positive surface EF trends, due to decreased SH and increased LH. There could also be a latitudinal dependency to the EF trends, reflecting differences related to the proximity to the Arctic Ocean, and/or varying evaporative regimes between the north and south portions of the study region^7^. Southern Siberia may be under a moisture-limited regime (i.e., EF a function of soil moisture) and northern Siberia may be energy-limited (EF a function of insolation). Increased July surface EF in the Central Siberian Plateau coincides with increased July afternoon precipitation. Results from a logistic regression model suggest a potential link or coupling between morning surface EF and the probability of afternoon precipitation. Significant increasing trends in July surface EF, July precipitation, and the probability of afternoon precipitation occurred preferentially over continuous permafrost, with non-significant or negative trends found over isolated, sporadic, and discontinuous permafrost regions. Snow cover in the same general region of positive July surface EF trends showed earlier snow melt in May and June, potentially providing a source of moisture for the enhanced LH and local moisture recycling in the region. Additional considerations involve reduced snow cover and increased temperatures potentially enhancing vegetation activity, thereby also resulting in more latent heating. Further work is also needed to better clarify the contributions of SH versus LH to positive EF trends in north-central Siberia where a larger decrease in SH was observed, relative to LH.

The results presented in this study are similar to those of previous land-atmosphere interaction studies in other parts of the globe^6^,^34^,^35^. However, this study examines the connection between surface EF and the atmosphere in a high latitude area. Eurasia in particular has experienced significant surface and subsurface changes, which likely affect the surface moisture supply for this potential land-atmosphere feedback. We find significant increases in surface EF, enhanced during the summer season. These trends are combined with significant connections between EF and the probability of precipitation. The implication of our findings is that as climate changes and permafrost and snow cover continue to degrade, these changes can contribute significantly to the hydrologic cycle in frozen ground regions. Climate models project intensification of the Arctic hydrologic cycle in response to climatic warming, manifest in terms of increased water fluxes between the land, atmosphere, and ocean^35^. However, as climate change causes continuous permafrost zones to transition to discontinuous, discontinuous to sporadic, sporadic to isolated, and isolated permafrost pockets to disappear, this will also alter the patterns of local moisture, atmospheric convection, moisture recycling, and hence the hydrologic cycle in high-latitude land areas. Rather than uniformly intensifying, concurrent changes in frozen ground distribution may decrease or ultimately even offset and counter any intensification in the Arctic hydrologic cycle^36^.

## Additional Information

**How to cite this article**: Ford, T. W. and Frauenfeld, O. W. Surface-Atmosphere Moisture Interactions in the Frozen Ground Regions of Eurasia. *Sci. Rep.* **6**, 19163; doi: 10.1038/srep19163 (2016).

## Supplementary Material

Supplementary Information
