# Supplementary material for: Surface–Atmosphere Moisture Interactions in the Frozen Ground Regions of Eurasia
Source: Sci Rep. 2016 Jan 18;6:19163. doi: 10.1038/srep19163 (PMC4726001; doi:10.1038/srep19163)
Supplement: Supplementary Information [file srep19163-s1.pdf]

Supplementary Information

Surface–Atmosphere Moisture Interactions in the Frozen Ground Regions of Eurasia

***Trent W. Ford[1] and Oliver W. Frauenfeld[2]**

[1]Department of Geography and Environmental Resources, Southern Illinois University, Carbondale, IL 62901

[2]Department of Geography, Texas A&M University, College Station, TX 77843-3147

The supplementary information includes Supplementary Figures S1 – S3. These figures display the spatial patterns of auxiliary datasets used in this study. Figure S1 shows the distribution of permafrost classes, based on the PZI, across the study region. Maps displayed in Figure S2 display the 1979-2012 surface EF climatology for the months January, April, July, and October. Finally, Figure S3 shows the average July 1st snow depth (cm) present on July 1st, taken over the period 1979-2012.

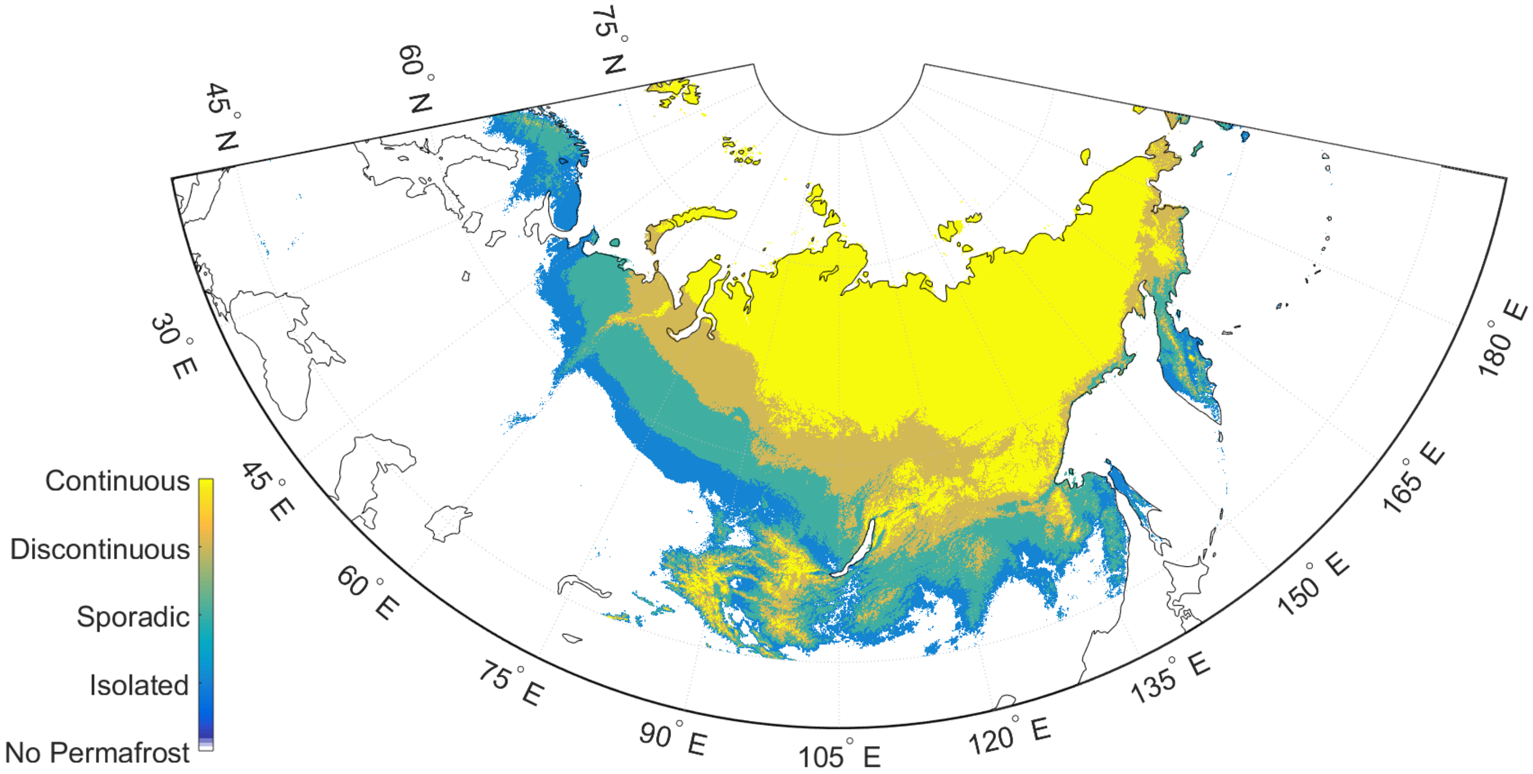

Supplementary Figure S1. Permafrost extent based on Permafrost Zonation Index. Grid cells are colored based on their permafrost zone category: continuous, discontinuous, sporadic, isolated, or no permafrost. The map was generated using Matlab software.

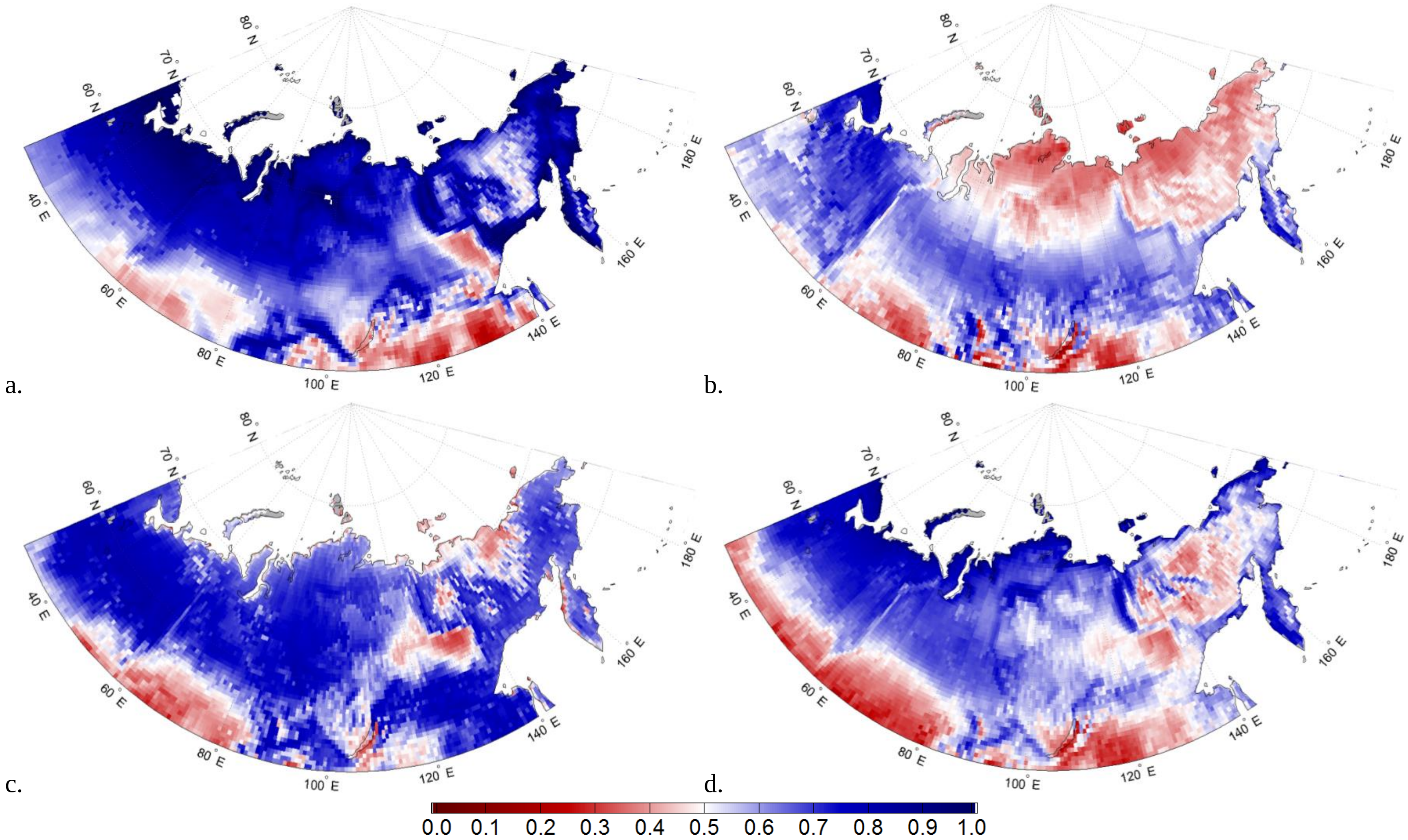

Supplementary Figure S2. 1979–2012 surface EF climatology from monthly MERRA-Land for (a) January, (b) April, (c) July, and (d) October. Maps were generated using Matlab software.

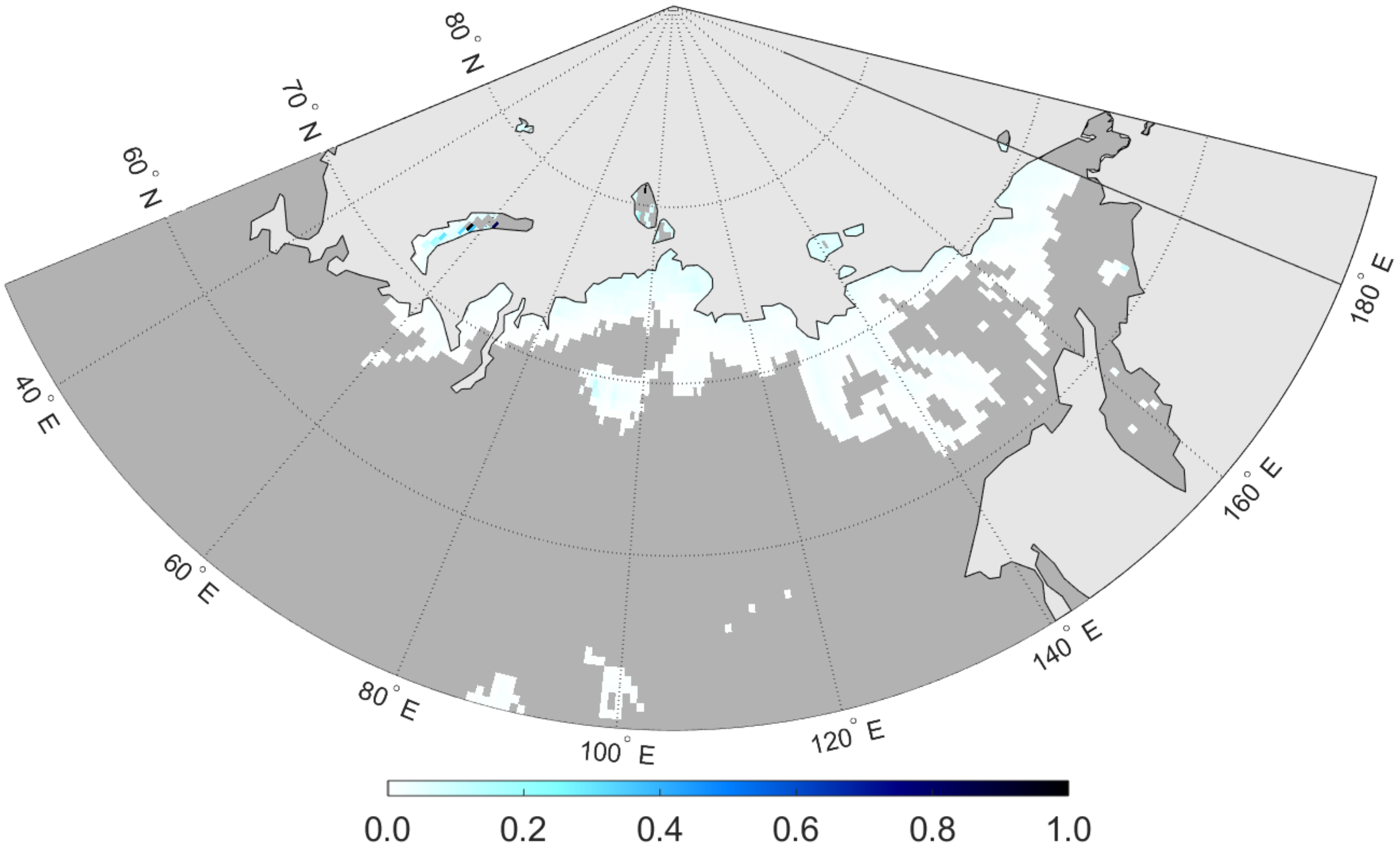

Supplementary Figure S3. Mean July 1st snow depth (cm).
